# Beyond the funding gap: Somalia’s aid shock is becoming a primary health-care crisis

**DOI:** 10.1186/s41182-026-01086-3

**Published:** 2026-09-28

**Authors:** Fardowso Mohamed Harbi, Ilyas Abdullahi khalif, Samar Yusuf Omar, Ahmed Abdinasir Abdulle

**Affiliations:** 1 Faculty of Health Sciences and Tropical Medicine, Somali National University, Mogadishu, Somalia; 2 Center for Health Research and Innovation, Somali National University, Mogadishu, Somalia

**Keywords:** Aid dependency, Primary health care, Health-system resilience, Health equity, Somalia

## Abstract

Somalia’s escalating humanitarian financing crisis is exposing a neglected dimension of health-system vulnerability: aid volatility can itself become a determinant of primary health-care (PHC) failure. Recent funding contractions have disrupted hospitals, primary facilities, mobile teams, and nutrition services while drought, displacement, infectious-disease outbreaks, and undernutrition continue to intensify health needs. In a system heavily reliant on external financing, abrupt donor withdrawal can destabilise frontline staffing, essential medicines, referral pathways, surveillance, and continuity of care. These disruptions disproportionately threaten populations already experiencing structural disadvantage, particularly children, displaced communities, and households exposed to recurrent climate shocks. We argue that humanitarian financing should, therefore, be understood not only as emergency assistance but also as a critical determinant of health-system resilience and equity. Protecting essential PHC functions, strengthening government stewardship, establishing predictable transition financing, and investing in climate-resilient community health systems are essential to prevent financing shocks from becoming enduring public-health crises.

**Dear Editor,**

Abrupt aid contraction in Somalia is no longer only a financing problem; it is interrupting primary health-care continuity in ways that weaken nutrition response, outbreak control, and day-to-day access to essential services.

The immediate signal is service closure. In 2025, funding reductions in Somalia resulted in the suspension of health services across eight hospitals, 40 primary health facilities, and 16 mobile teams in 21 districts, affecting approximately 350,000 people, according to humanitarian situation reports from WHO and health-sector partners [1]. During the same period, more than 300 nutrition centres were closed and food, health, and WASH services were reduced, increasing vulnerability among affected populations [1]. These events matter scientifically, because Somalia’s health system already has among the world’s lowest public-health financing levels and remains heavily aid-dependent [2, 3].

Somalia’s health financing structure remains highly dependent on external assistance. The Health Sector Resource Mapping and Expenditure Tracking exercise conducted by the Federal Ministry of Health and partners showed that donor resources constitute a major component of health-sector financing, while domestic government contributions remain limited [4]. This dependence increases vulnerability to abrupt aid reductions, because disruptions in external financing may affect the continuity of essential primary health-care functions. When external financing is reduced abruptly, the consequences extend beyond programme reductions. Evidence from fragile and conflict-affected settings suggests that financing disruptions may affect workforce stability, essential medicine availability, referral pathways, surveillance capacity, and continuity of routine services [5]. However, the specific effects of recent funding reductions on these health-system functions in Somalia require further empirical assessment. Unlike chronic underfunding, aid volatility creates uncertainty, because sudden and unpredictable financing changes can disrupt the ability of health systems to maintain essential functions. In fragile and conflict-affected settings, such as Somalia, where international assistance supports critical primary health-care activities, unstable funding environments can threaten continuity of care, health-system planning, and resilience [3]. Therefore, aid volatility should be considered a distinct health-system determinant, because its effects arise not only from reduced resources but also from instability in the financing environment. Somalia’s facilities already struggle with essential medicines, water, electricity, and weak regulation of supply chains, so financing shocks propagate rapidly across the whole PHC platform [3]. Broader resilience evidence from fragile and conflict-affected settings shows that short-term humanitarian funding cycles, fragmented governance, and donor-dominated decision-making undermine continuity and local ownership, while more predictable humanitarian–development–peace approaches improve coherence and systems strengthening [5, 6].

The consequences are most visible, where climate, displacement, infection, and undernutrition interact. Somalia’s recurrent droughts and floods intensify food insecurity, displacement, poor WASH access, and outbreaks of cholera, acute watery diarrhoea, measles, and diphtheria [7]. Among displaced populations, severe malnutrition, respiratory infections, and diarrhoeal disease are leading causes of death, and among children under five, malnutrition, measles, and neonatal pneumonia predominate [8]. Earlier Somali cohort evidence likewise found high child mortality in IDP camps, with diarrhoeal disease, measles, and severe malnutrition among the leading causes, underscoring what is put at risk when frontline PHC, immunisation, nutrition, and WASH-linked services falter [9]. Aid volatility is particularly consequential in this context, because financing contractions may occur during periods when climate-related health needs are increasing. In Somalia, droughts, floods, displacement, and disease outbreaks generate sudden increases in demand for primary health-care services, including nutrition support, disease surveillance, immunisation, and community-based care [7]. When financing becomes unpredictable during these periods, PHC systems may have reduced capacity to absorb these surges and maintain essential services. The interaction between climate-related shocks and unstable financing, therefore, represents a compounded challenge for health-system resilience [8].

Current responses remain too episodic. During acute funding contractions, priority should be given to protecting essential PHC functions that prevent immediate service collapse, including essential medicines, community health workers, referral mechanisms, and core surveillance activities. These short-term continuity measures should be complemented by longer-term health-system reforms, including transition financing arrangements, integration of essential health workers into sustainable government structures, stronger domestic health financing, and investment in climate-resilient PHC systems. Donors and UN agencies should support predictable financing, coordinated planning, and alignment with national priorities, while Somali federal and state authorities should strengthen stewardship, accountability, and integration of externally supported services into government-led health strategies [2].

Although this Letter focuses on Somalia, the underlying challenge applies to other highly aid-dependent fragile and conflict-affected health systems, where external resources support essential health functions. Countries facing similar vulnerabilities may experience comparable risks when humanitarian financing declines without adequate transition planning or domestic capacity to maintain services. The Somali experience, therefore, highlights the importance of predictable financing arrangements, stronger national stewardship, and resilience-oriented approaches to protect PHC continuity during periods of external funding uncertainty.

## Data Availability

No datasets were generated or analysed during the current study.
